# A Unique Case of Minor Surgery

**Published:** 1886-11

**Authors:** W. A. Barton

**Affiliations:** Newport, Texas


### A UNIQUE CASE IN MINOR SURGERY.

*Editor Daniel's Texas Medical Journal:*

HAVING had recently a rather unique case in minor surgery, I thought to give a sketch in your valuable Journal.

Mrs. J. K. applied for advice in regard to a foreign body, which she thought to be a pin or needle, which made its appearance some ten months since, in the left mammal region. I found the body located in the fifth intercostal space, lying obliquely with point directed downward and outward, with head deeply imbedded in superficial facia. As the point lay immediately beneath the integument, I thought, to make an incision just over it and use small forceps, would be all that was necessary; but I found it almost impossible to get a hold on it, it being in the loose fascia. Having gained the patient's consent, I concluded to use an anæsthetic. I then had Dr. Welsh called, who came, accompanied by Dr. Burk, both of this place. They agreeing with me, we at once proceeded. After getting a good state of anæsthesia, we made an incision the length of the foreign body, cutting well down and separating the gristly cyst in which it was incased.

It was found to be an iron pin, very much corroded, so much so that it came out in three pieces, which, being placed together, gave the outline of the pin. The incision was immediately closed by two or three interrupted sutures, and the wound dressed antisceptically.

I have written these few lines for information, as the patient could furnish no clue. She does not recollect to have smallowed a pin or to have stuck one in her person. If anyone feels disposed to give a few suppositions, or ideas, in regard to it, they will be thankfully received.

W. A. BARTON, M. D.
Newport, Texas.

